# Dr. George P. Eddy

**Published:** 1916-10

**Authors:** 


**Dr. George P. Eddy,** Buffalo 1865, died suddenly at his home in Lewiston, July 4, aged 75.

